# Acute kidney injury and renal angina

**DOI:** 10.12861/jrip.2013.12

**Published:** 2013-06-01

**Authors:** Alaleh Gheissari

**Affiliations:** ^1^Isfahan Child Growth and Development Research Center, Isfahan University of Medical Sciences, Isfahan, Iran

**Keywords:** Acute kidney injury, Chronic kidney disease, Renal angina

**Implication for health policy/practice/research/medical education:**

Recently, the term renal angina (RA) has been described to straighten using biomarkers in at-risk patients who have a combination of illness severity/risk and even small changes in kidney function. RA is a valuable guideline to identify high risk patients and improve the positive predictive value of serum and urine biomarkers to predict early stages of AKI and its severity.

Acute kidney injury (AKI), previously termed as acute renal failure is not uncommon among critically ill pediatric patients. A recent survey demonstrated a twenty-fold increase in AKI rates from 0.5 to 9.9 cases per 1000 hospitalized children between 1982 to 2004 (1). AKI has been reported as an independent risk factor for mortality (2-4). A cross talk among kidney, lung, brain and heart may raise harmful and deadly consequences of AKI in critically ill patients (5-6). In addition, non-renal complications in patients such as tendency to bleeding, severe infections and sepsis are partly responsible for high mortality rate in AKI in ICU setting (7). Increasing serum creatinine or even using severity of illness (SOI) scoring systems and organ dysfunction scores (OD) including: APACHE (Acute Physiology and Chronic Health Evaluation); SAPS (Simplified Acute Physiological Score); PRISM (pediatric Risk of Mortality) are not helpful in finding patients at risk of AKI (8,9). Therefore, new biomarkers have been proposed widely to predict early stages and progression of AKI (10). Indeed, the prognosis of AKI biomarker ‘positive’ but serum creatinine ‘negative’ AKI is equal to classical functional AKI (11). Among numerous under-studied biomarkers; neutrophil gelatinase-associated lipocalin (NGAL), interleukin 18 (IL-18), Kidney Injury Molecule-1 (KIM-1) and Liver Fatty Acid Binding Protein (L-FABP) have been investigated extensively (12). Recently, the term renal angina (RA) has been described to straighten using biomarkers in at-risk patients who have a combination of illness severity/risk and even small changes in kidney function (10,13). RA is a valuable guideline to identify high risk patients and improve the positive predictive value of serum and urine biomarkers to predict early stages of AKI and its severity (10). Basu *et al*. proposed a simple equation to estimate RA (10):

Renal angina threshold= risk of AKI× evidence of AKI

Appearing the clinical evidences of overt AKI will decrease the requirement to fulfill the criteria of RA. It has been proposed that completing pediatric RA criteria is based on merging initial risk stratification with early signs of renal dysfunction (small changes in serum creatinine or mild degrees of fluid accumulation) (10). More studies are needed to validate RA to optimize biomarker measurement in critically ill patients.

## Author’s contribution

AG is the single author of the manuscript.

## Conflict of interests

The author declared no competing interests.

## Ethical considerations

Ethical issues (including plagiarism, misconduct, data fabrication, falsification, double publication or submission, redundancy) have been completely observed by the authors.

## Funding/Support

None.
